# PICO-based assessment and categorization of evidence for digital health interventions: an inductive framework development

**DOI:** 10.3389/fdgth.2026.1755598

**Published:** 2026-02-18

**Authors:** Uwe Buddrus, Jan-Oliver Kutza, Johannes Thye, Moritz Esdar, Ursula Hertha Hübner, Jan-David Liebe

**Affiliations:** 1 Digital Society, Research Centre for Health and Social Informatics, School of Business Management and Social Sciences, Hochschule Osnabrück—University of Applied Sciences, Osnabrück, Germany; 2 Geschäftsbereich III—Digitalisierung und EHealth, Deutsche Krankenhausgesellschaft e.V., Berlin, Germany; 3 Institute of Medical Informatics, UMIT TIROL—Private University for Health Sciences and Health Technology, Hall, Austria

**Keywords:** assessment, categorization, classification, digital health interventions, evidence, framework, outcomes, systematic reviews

## Abstract

**Background:**

Despite the increasing number of systematic reviews on digital health interventions (DHIs), clear and robust evidence remains elusive due to methodological shortcomings in formulating research questions and conducting search and screening processes. The growing volume of reviews necessitates higher-level syntheses like umbrella reviews and evidence gap maps, requiring methods for rapid, systematic evidence assessment at the abstract level.

**Objective:**

With the development of the PICO-based Assessment and Categorization of Evidence for Digital Health Interventions (PACE4DHI) framework we aim to enable the efficient structured screening of systematic reviews and meta-analyses at the level of abstracts for subsequent evidence and gap mapping (EGM).

**Methods:**

A comprehensive literature search was performed across five databases, adhering to PRISMA guidelines, to capture systematic reviews and meta-analyses published between 2011 and October 2023. All categories of DHIs, populations, settings, and outcomes were considered. From 21,161 results, we screened 9,030 titles and abstracts post-de-duplication, with 2,528 remaining. To construct the framework, thematic analysis was conducted on a random sample of 250 studies. The framework's accuracy was validated on 138 open-access articles through full-text comparisons.

**Results:**

The PACE4DHI framework encompasses 41 categories, spanning 11 problems (e.g., cardiovascular diseases), 13 DHIs (e.g., telemedicine), 6 comparative care settings (e.g., outpatient care), 7 outcome dimensions (e.g., effectiveness), and 4 evidence classification levels. The PICO-categorization and evidence classification was confirmed with varying accuracy and largely consistent results at both abstract and full-text levels. Variability in the accuracy reflects that abstracts provided more detail on problems and interventions than they did for the comparator and outcomes. The likelihood of conclusive evidence was more accurately predicted for cardinal classes (high and low) than for inconclusiveness.

**Conclusions:**

The PACE4DHI framework provides a systematic and pragmatic methodology, with potential to enhance structured access to existing evidence. The framework may also inform the research questions and the search and screening strategies of future systematic reviews. The application in EGM has potential to optimize evidence-based decision-making, while also enabling precise identification of research gaps. Its use with artificial intelligence tools may facilitate efficient ongoing evidence screening and synthesis, ultimately supporting a searchable evidence database.

## 1 Introduction

Digitalization in healthcare has led to a steady interest in researching the impacts of Digital Health Interventions (DHIs). Various digitalization programs for national healthcare systems have been initiated and implemented in recent years by governments with the overarching objectives to improve the delivery and quality of care, efficiency and cost-effectiveness. These developments are accompanied by a broad and exponentially growing body of evidence on the impact of DHI, often summarized in systematic reviews. A bibliometric analysis of digital health and mobile health related global research publications by Aagja et al. indicates a significant increase in the number of publications starting in 2017 (1). Likewise, over the last two decades there has been an exponential rise in the number of systematic reviews published across nearly every discipline (2–4).

Despite the growing number of studies, it often remains unclear which specific effects of digital health interventions (DHIs) are robustly supported. The main reasons are imprecisely formulated research questions, ineffective search and screening strategies, and fragmented databases (5). At the same time, the increasing number of systematic reviews, many of which are redundant or overlapping, hampers the recognition of reliable evidence for policymakers and practitioners (3).

To address this problem, the consistent use of structured frameworks such as PICO can help to formulate answerable intervention questions, derive appropriate search terms, and improve the identification of relevant evidence (5–10).

Building on this, higher-level syntheses such as umbrella reviews and evidence and gap maps (EGMs) provide systematic ways to facilitate informed and evidence-based decision making and identify key research gaps (11–13). While EGMs are already established in other health domains (14, 15), with just one identified to date (16, 17), there remains a clear need for systematically developed EGMs focusing on DHIs.

For both, umbrella reviews and EGM, large numbers of abstracts must be efficiently and systematically screened regarding clear indications of the presence and strength of evidence, or its absence, related to problems/populations, interventions, care settings and outcomes of interest.

The aim of this study is to develop a framework for the PICO-based Assessment and Categorization of Evidence for Digital Health Interventions (PACE4DHI) based on abstracts of systematic reviews and meta-analyses. PACE4DHI shall leverage structured categorization aligned with current research and established definitions to facilitate efficient abstract screening and subsequent continuous EGM. Against this background this study addresses the following research questions:

1. What are the most prevalent categories based on the PICO scheme to systematically structure current research on DHIs?
2. What are the key criteria to accurately classify the likelihood of finding conclusive evidence in full texts during the title and abstract screening process?

## 2 Materials and methods

### 2.1 Study design

An inductive approach, inspired by the methodological process of umbrella reviews, was used to develop the PACE4DHI framework. We conducted the study in line with the JBI Manual for Evidence Synthesis (18), with the aim to clarify and identify definitions, characteristics or factors related to our concept.

### 2.2 Search strategy

The search strategy was developed with the aim of including existing systematic reviews and meta-analyses which address (a) a broad range of DHIs and (b) a broad range of potential associated outcomes in (c) any healthcare setting and d) population.

A total of 33 DHI-related and 41 outcome-related search terms were developed by the research team (UB, ME, OK, UH, JL), and, depending on the database searched, applied as MeSH-terms to subject headings or as plain search terms on titles and abstracts. A complete documentation of all search terms is available in Supplementary Material 1. A comprehensive literature search was conducted for the timeframe 2011 to October 2023. To capture the heterogeneity of care settings and DHI application scenarios, the search included all areas of health care. These range from inpatient hospital care through outpatient and community-based care to self-care, without any geographic boundaries. The search was limited to systematic reviews or meta-analyses published in English or German. We searched five leading databases: Scopus, AISeL, EBSCO/CINAHL, Cochrane Library and MEDLINE via PubMed.

### 2.3 Study selection

The search results were uploaded to Covidence, a software for managing and streamlining systematic reviews. All potential studies for further analyses such as evidence and gap mapping (EGM) and systematic thematic umbrella reviews were screened by UB, OK, JT and ME and cross-checked by JL and UH to determine if the title and abstract fulfilled the pre-defined inclusion and exclusion criteria. A complete documentation of all inclusion and exclusion criteria is available in Supplementary Material 2.

The initial abstract review and screening process was completed in February 2024. After all studies were screened, all eligible studies were exported, additional duplicates were identified, checked and omitted and a random sample was drawn.

The sample size of 250 abstracts accommodates the need for a relatively large qualitative sample to achieve thematic saturation and category stability in the thematic analysis, given the heterogeneity of digital health interventions, populations, settings and outcomes.

A formal risk-of-bias or quality appraisal was not performed because the objective was abstract-level categorization and framework development, not effect estimation. Also, inconsistent reporting in abstracts would have prevented appraisal.

### 2.4 Data extraction

In line with our research objective, a set of data extraction fields and variables were developed. Beside meta-information, these were the PICO-elements, i.e., Problem and Population, Intervention, Comparator and Outcome, as well as the results and conclusions, representing the likely scope of evidence. All information was extracted at the title and abstract level. A complete documentation of the sample with all extracted data and analyses is available in Supplementary Material 3.

### 2.5 Data analysis

#### 2.5.1 Thematic analysis

Results were synthesized using descriptive and thematic synthesis methods. No statistical synthesis was performed. Thematic analysis was performed to derive an initial basis for a systematic categorization of PICO-elements as well as the classification of the likelihood to derive conclusive evidence from the full texts. For the thematic analysis Braun and Clarke's six phase framework (19) was applied. Phase 1 (familiarizing) was conducted by organizing abstracts according to the level of PICO-specification and extracting results and conclusions. In phase 2 (initial coding), relevant information related to each PICO-element and to the reporting of results, quantification and direction of effects, and conclusions was isolated. Phase 3 (searching for themes) involved initial secondary research on existing approaches to the categorization of each PICO-element as well as criteria for the conclusiveness of evidence. Themes describing the PICO-elements and evidence were generated by allocating the coded information to the initially identified themes for categories and criteria and searching for additional themes. In phase 4 (reviewing themes), themes were iteratively refined until consensus was reached (UB, OK, JL). In phase 5 (defining themes), themes were named and defined (UB, JL). The final categories and classification criteria included in the framework were discussed and discrepancies were resolved with JL serving as thematic auditor. Phase 6 (writing) was completed with this article. A complete documentation of the thematic analyses is available in Supplementary Material 3, while Supplementary Material 4 provides Tables 2–5 with the thematic categories of PICO-elements including references.

#### 2.5.2 Validation analysis

For the internal validation, 138 open access articles providing full texts were identified from the sample of 250 studies for rapid full text review to test the accuracy of the proposed abstract-based categorization and classification. The use of this availability-based subsample was a pragmatic decision. Details are available in Supplementary Material 3—sheets PICO Validation and Evidence conclusiveness.

## 3 Results

### 3.1 PRISMA flowchart and study characteristics

The initial electronic database search yielded a total of 21.161 results, of which 12.131 duplicates were automatically identified by Covidence, and an additional 100 duplicates were identified manually. A total of 9.030 Titles and Abstracts were screened, and 6.502 studies excluded as irrelevant since not fulfilling the defined inclusion criteria. Of the remaining 2.528 studies, a random sample of 250 was selected for the thematic analysis and 138 of these were included in the validation analysis.

**Figure 1:**
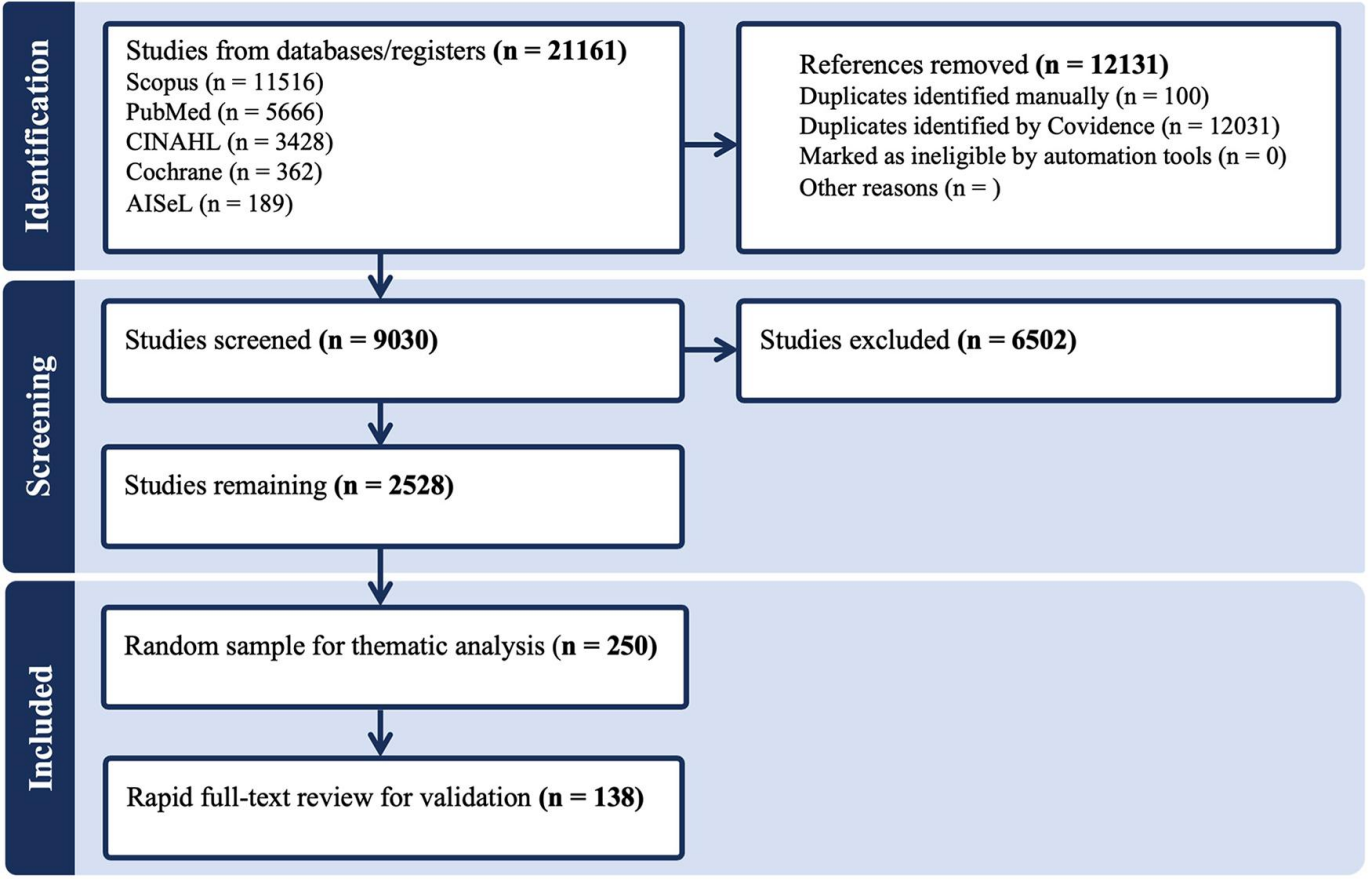
PRISMA flowchart.

Figure 1 shows the PRISMA flowchart.

Key study characteristics in the samples for framework development and validation are presented in Table 1.

**Table 1:** Study design and publication year in the random samples, number (*n*) and percentage (%) of *N* = 250 abstracts.

Study characteristics	Development *n* (%)	Validation *n* (%)
Study design	**250 (100.0)**	**138** (**100.0)**
Systematic review	146 (58.4)	80 (58.0)
Systematic review and meta-analysis	58 (23.2)	37 (26.8)
Systematic literature review	23 (9.2)	7 (5.1)
Meta-analysis	13 (5.2)	7 (5.1)
Systematic review of (systematic) reviews	6 (2.4)	4 (2.9)
Systematic review of systematic reviews and meta-analyses	4 (1.6)	3 (2.2)
Publication year	**250 (100.0)**	**138** (**100.0)**
2023 (published before November 2023)	42 (16.8)	21 (15.2)
2022	59 (23.6)	32 (23.2)
2021	32 (12.8)	18 (13.0)
2020	21 (8.4)	13 (9.4)
2012-2019	96 (38.4)	54 (39.1)

### 3.2 Systematic categorization of PICO-elements (research question 1)

#### 3.2.1 P—problem/patient/population

“P” was found to be mostly described in terms of a disease, disorder or condition.

The basis for the categorization of the problem were the six most common chronic or noncommunicable diseases (*N*CDs), as described for example by the WHO (20) or Robert Koch Institute (21), i.e., *cardiovascular diseases (CVDs), cancers, diabetes, mental health issues, diseases of the musculoskeletal system and chronic pulmonary diseases, especially asthma and COPD*. The WHO identifies tobacco use, physical inactivity, the harmful use of alcohol, and unhealthy diets as major factors increasing the risk of dying from an NCD (20). These factors were categorized as *behavior.* Other sources including the Centers for Disease Control and Prevention (CDC) and National Council on Aging (NCOA) (22, 23) additionally list stroke, Alzheimer's disease and dementia, which were categorized as *neurological disorders* (24), although stroke may also be categorized as a cardiovascular disease (CVD) (25).

In the thematic analysis, these eight categories occurred in about two thirds of all studies (193/250, 63.7%). For the remaining third, we defined three additional thematic categories covering *diverse specific conditions* that were investigated in less than three studies*, diverse groups of conditions* that encompass more than three of the other categories*,* and *diverse, unspecified* for studies without focus on any medical condition or specialty.

Table 2 provides an overview of the thematic categorization focusing on the various problems (P) described in the sample abstracts.

**Table 2:** Thematic categories of PICO-element P—problem, number (*n*) and percentage (%) of *N* = 250 abstracts, multiple allocations possible.

#	Category	Description and examples	*n* (%)
1.	Cardiovascular diseases (CVDs)	Includes heart failure, high blood pressure/hypertension, stroke, ischemic heart disease, coronary artery disease, cardiac and vascular surgery, cardiac rehabilitation.	41 (16.4)
2.	Diabetes	Any studies related to diabetes in general, specific types (mellitus, type 2), diabetes-related conditions, and diabetes as a risk factor.	30 (12.0)
3.	Behavior	Concerned with changing lifestyle behaviors including physical activity, diet and weight, smoking cessation, contraception, etc.	28 (11.2)
4.	Cancers	Encompasses various forms of cancers, cancer screening and detection, cancer-related conditions (e.g., polyps, hematological conditions), cancer treatment, burden, and aftercare.	26 (10.4)
5.	Mental health	Encompasses various mental health issues and mental illnesses, including depression, suicidal ideation, insomnia, caregiver stress.	25 (10.0)
6.	Neurological disorders	Any studies on disorders that affect the brain, spinal cord or nerves, and related interventions, including dementia, stroke, Alzheimer's, autism, spinal cord injury, neurosurgery, neurorehabilitation.	25 (10.0)
7.	Musculoskeletal disorders	Any studies on disorders that affect muscles, tendons, joints and cartilage, including rheumatic diseases such as osteoarthritis, related pain, and rehabilitation following arthroplasty.	10 (4.0)
8.	Chronic pulmonary diseases	Particular focus on asthma and COPD or among the identified conditions targeted by the DHI.	8 (3.2)
9.	Diverse groups of conditions	Encompasses unspecified groups of conditions such as chronic diseases, NCDs, chronic wounds, digestive diseases, and medical specialties, e.g., obstetrics, pediatrics, palliative care, urology, etc.	40 (16.0)
10.	Diverse, unspecified	Studies with no specific focus on any medical condition or applicable across all health conditions; often related to medication.	27 (10.8)
11.	Diverse specific conditions	Includes specific diseases (e.g., HIV, COVID-19, Tuberculosis), diagnostic, therapeutic or surgical interventions (e.g., Colon Capsule Endoscopy, renal transplant) and clinical objectives (e.g., prediction of hospital-acquired pressure injuries).	17 (6.8)
	Multiple category allocation	Studies may be allocated to more than one category for stroke as CVD and neurological disorder, for comorbidities such as mental health issues associated with cancer, and for unspecified or wider research scope (e.g., chronic diseases, NCDs) but focused findings (e.g., diabetes, CVDs).	23 (9.2)

#### 3.2.2 I—intervention—technology

“I” refers to the digital health interventions and their underlying technologies.

Secondary research did not identify a suitable basis for the categorization of DHIs, nor deliver consistent definitions. Consequently, the intervention technologies were inductively identified from the abstracts and, through thematic analysis, systematically categorized according to consistencies in existing definitions, while aiming to ensure clear differentiation among categories.

In secondary sources, we found a plethora of definitions for digital health, which most commonly encompassed mHealth and eHealth, but were often related to various other concepts such as telehealth (167). There appeared to be a particular lack of consensus on the concept and definition of eHealth (168–170). The term mHealth was defined more precisely overall (171, 172), yet there was some overlap with other concepts, especially eHealth, telehealth, telemedicine and telerehabilitation. However, telehealth, telemedicine (173) and telerehabilitation (174) were relatively well and consistently defined and could be distinctly differentiated from mHealth by their intervention focus and apparent health provider interaction.

In the abstracts, we found the terms digital health, eHealth, mHealth, remote, web-based or online interventions, but also telehealth, telemedicine and telerehabilitation were sometimes interchangeably or misleadingly used by researchers and study authors. As a result, we categorized some “mHealth” studies as telehealth, telemedicine and/or telerehabilitation (29, 106, 122, 130, 175, 176). Similarly, some “telehealth” studies were categorized as telemedicine and/or telerehabilitation (32, 120, 135, 150, 177–179).

Thematic analysis of the random sample identified 11 categories to which at least three studies could be allocated, and two additional themes that may represent preliminary categories.

Table 3 provides an overview of the thematic categorization focusing on the various interventions (I) described in the sample abstracts.

**Table 3:** Thematic categories of PICO-element I—intervention, number (n) and percentage (%) of *N* = 250 abstracts, multiple allocations possible.

#	Category	Description and examples	*n* and %
1.	Telemedicine	Refers to remote patient interaction with clinical providers for assessment, diagnosis, consultation, therapy and management, including telemonitoring, and specialty forms such as telepsychiatry, teledermatology, telerheumatology, telenursing, telestroke, etc	66 (26.4)
2.	eHealth/Telehealth	Includes remote and online web- or internet-based interventions via home computers or mobile devices without or with interaction (i.e., telehealth), and with a focus on behavior change, incl. CBT, health education, counseling, and support for self-management	64 (25.6)
3.	mHealth	Focuses on self-care and self-management without health provider interaction, involving apps, wearables and sensors	60 (24.0)
4.	Artificial Intelligence (AI/ML)	Encompasses studies on AI techniques, including deep learning and machine learning, used in diagnostics, detection, prognosis and prediction, and treatment delivery, e.g., by AI-powered chatbots	30 (12.0)
5.	Telerehabilitation	Concerns the delivery of rehabilitative programs and exercise interventions via videoconferencing, apps, and virtual reality rehabilitation. Includes cardiac and neurorehabilitation	29 (11.6)
6.	Medication management	Encompasses a variety of technologies and processes, from medication reconciliation, through electronic prescribing and medication administration, to supporting adherence	14 (5.6)
7.	EHR/EMR/EPR/HIE	Covers Electronic Health Records (EHRs), Electronic Medical Records (EMRs), Electronic Patient Records (EPRs), and Health Information Exchange (HIE) between them	13 (5.2)
8.	Clinical Decision Support (CDS)	Relates to systems aiding clinical decision-making, including prediction and evidence-based treatment, also using AI and ML	13 (5.2)
9.	Robotics	Encompasses any use of robotic technologies in healthcare, including surgeries, treatment, and logistics, e.g., robot-assisted surgery, robot-assisted gait training, social or companion robots	6 (2.4)
10.	Virtual Reality (VR)	Encompasses a wide range of applications such as simulation, visualization, training, teaching, learning, etc., e.g., utilized for treatment compliance and for rehabilitation training	4 (1.6)
11.	CPOE	Computerized Physician Order Entry systems	3 (1.2)
12.	Other themes	The examples pertain to PACS, wound imaging, and web-based medical appointment systems and may constitute the additional categories “Imaging” and ‘Patient Portal’	3 (1.2)
	Multiple category allocation	Most often pertaining to intersections between mHealth, eHealth/telehealth, telemedicine, and telerehabilitation, along with combinations relating to CDS and AI or CPOE	44 (17.6)

#### 3.2.3 C—comparison—healthcare setting

“C” is here intended to reflect the healthcare settings to which the DHIs primarily compare or apply.

The basis for the categorization was identified and pre-defined from listings of healthcare settings at various levels of detail and with varying overlaps (232–234). Most common across secondary sources were *hospital* (emergency and inpatient care), *outpatient* (primary and specialist care practices and clinics), *rehabilitation* centers, and long-term care facilities (e.g., nursing homes). Residential long-term care and many of the diverse remaining settings, e.g., birth centers, mental health facilities, hospices, pharmacies, were categorized as *community-based care*. Community care also covers a wide range of additional health services including health promotion, occupational therapy, palliative care, physiotherapy, speech and language therapy, etc. (235). Other healthcare facilities were allocated to one of the before-mentioned categories, e.g., urgent care centers (236) and outpatient surgery centers to outpatient care. In line with some sources, we considered *home-based self-care* as a separate setting (232, 233).

Imaging and radiology centers, laboratories and pathology services represent healthcare facilities that do not directly provide care to patients but facilitate diagnoses by other clinicians. Thus, we added “*intersectoral*” to reflect that some studies investigate interventions or conditions requiring or focusing on health information exchange or a multidisciplinary approach between care providers.

Using the pre-defined categories and applying implicit rules when no setting was explicitly specified, we were able to allocate a category to each abstract.

Table 4 provides an overview of the thematic categorization focusing on the various comparative settings (C) described in the sample abstracts.

**Table 4:** Thematic categories of PICO-element C—comparison, number (n) and percentage (%) of *N* = 250 abstracts, multiple allocations possible.

#	Category	Description and examples	*n* and %
1.	Outpatient care	Explicitly encompasses primary care, ambulatory care, outpatient setting, non-urgent health care services/practices, and a variety of typical outpatient specialist care and mental health services. Implicitly referred to by the primary management of many chronic conditions, e.g., diabetes, CVDs, COPD, chronic wounds	83 (33.2)
2.	Self-care and self-management	Focuses on interventions to improve self-care and self-management skills in various conditions, self-support for lifestyle behavior changes and for informal caregivers, typically without provider involvement, and often linked to mHealth	66 (26.4)
3.	Community care	Includes behavioral and psychosocial specialists, physical therapy, home-based, community and residential care settings, long-term care, palliative care, and preventive health care	35 (14.0)
4.	Intersectoral care	Reflects interventions that transcend care settings, such as Health Information Exchange, medical data processing in EHRs (e.g., for screening), medical interpretation, a multidisciplinary care approach. Implicitly pertains to cancer diagnostics and treatment	32 (12.8)
5.	Hospital care	Refers to hospital inpatient care, acute care, surgery-related interventions, peri- and neonatal care, intensive care, emergency department visits; Implicitly pertains to CPOE or CDSS	31 (12.4)
6.	Rehabilitation	Covers explicit comparisons with conventional rehabilitation, in various rehabilitation settings, specifically cardiac rehabilitation, neurorehabilitation, and cancer aftercare	28 (11.2)
	Multiple category allocation	Studies applying to two or more settings, most often self-care and community care, inpatient hospital and outpatient care or outpatient care with any other settings	23 (9.2%)

#### 3.2.4 O—outcome

“O” refers to the anticipated results, impacts or effects of DHIs.

Outcomes were initially categorized based on the Six Domains of Healthcare Quality recommended by the IOM (now the National Academy of Medicine): *Patient safety*, *Effectiveness, Patient-centeredness, Timeliness, Efficiency, Equity/Access* (271).

We added “*Satisfaction*”, in accordance with Donabedian (272), and acknowledging that patient satisfaction is becoming more important in evaluating care quality (273), as confirmed by our thematic analysis.

Table 5 provides an overview of the thematic categorization focusing on the various outcome dimensions (O) described in the sample abstracts.

**Table 5:** Thematic categories of PICO-element O—outcome, number (*n*) and percentage (%) of *N* = 250 abstracts, multiple allocations possible.

#	Category	Description and examples	*n* and %
1.	Effectiveness	Encompasses a wide range of measurable clinical outcomes including symptoms (e.g., pain, depression), disease control indicators (e.g., blood pressure), mortality, biochemical indicators (e.g., glucose, HbA1c), anthropometric indicators (e.g., BMI, weight), physical function/exercise capacity (e.g., speed, balance, etc.), cognitive function (e.g., memory), pulmonary function, healing rate, obstetric outcomes, and certain process of care measures	151 (60.4)
2.	Patient-centeredness	Any outcomes related to an interventions responsiveness to patient preferences, needs, and values, with a focus on psychosocial and patient-centered implementation outcomes, including health-related quality of life (HRQoL) and well-being, lifestyle changes (e.g., diet, physical activity, abstinence, contraception), activities of daily living, emotional/social skills and needs, patient engagement and treatment adherence/compliance, education, self-management/-efficacy, informal caregiver support, and care coordination	119 (47.6)
3.	Efficiency	Covers cost-effectiveness, costs and cost-savings, healthcare resource utilization, e.g., emergency department visits, test consumption, hospitalizations, length of stay, and readmissions, efficiency of work practices, productivity and time savings, patient attendance and no-show rates	63 (25.2)
4	Patient Safety	Focuses on avoiding harm, including improved diagnostic decision making and accuracy in diagnoses, improved medication safety, prophylaxis, prediction and prevention of adverse events, complications, clinical deterioration and exacerbation, adverse pregnancy outcomes, procedural errors, infections, pressure injuries, thromboembolic events, hypoglycemia, falls, suicide, and provider adherence to guidelines and protocols	62 (24.8)
5.	Satisfaction	Comprises user satisfaction among patients and healthcare providers, related to satisfaction with the treatment and acceptability/acceptance of the intervention	37 (14.8)
6.	Timeliness	Relates to decreased waiting time, onset-to-door (OTD) duration, turnaround times, earlier diagnosis, timely intervention, time to clinical action; also, immediate access to special services	8 (3.2)
7.	Equitable Access to Care	Involves improved access to health care providers including specialist care, services and resources, but no study in the sample specifically reported on equitable access	6 (2.4)
	Multiple category allocation	Multiple outcomes are commonly reported on two or more (up to five) outcome dimensions	131 (52.4)

#### 3.2.5 Validation of the accuracy of the categorization of PICO-elements

A rapid full text review was conducted on 138 open access articles for which the full texts were available to test the accuracy of the abstract-based PICO-categorization.

We considered our categorization as confirmed, when the full text detailed the respective PICO-elements in the methodology and/or in results, e.g., in overview tables of included studies. Partial confirmation refers to either additional categories being identified or not all categories (i.e., fewer) being confirmed in the full text. Cases in which each category had to be changed were considered as not confirmed.

Table 6 provides an overview of the validation test results. Details are available in Supplementary Material 3.

**Table 6:** Accuracy of the categorization of PICO-elements, number of full texts (*N* = 138).

Accuracy	P	I	C	O
Confirmed	96.4% (133/138)	92.0% (127/138)	79.0% (109/138)	75.4% (104/138)
Partly confirmed	3.6% (5/138)	8.0% (11/138)	18.1% (25/138)	23.9% (33/138)
Not confirmed	0.0% (0/138)	0.0% (0/138)	2.9% (4/138)	0.7% (1/138)
Re-categorization n reason	5 additional	11 additional	24 additional 1 fewer 4 other setting	29 additional 4 fewer 1 other outcome

The variability in the accuracy reflects that the abstracts provided a clearer and more comprehensive description of the problems and interventions than they did of the comparator and outcomes.

Most partial confirmations related to additions of one or more categories that were identified at least for some included primary studies.

Rarely, categories were allocated wrongly—in only five cases for the comparative setting and outcomes, respectively. While four implicitly determined settings were not confirmed (e.g., categorized as self-care, but primary studies were conducted in outpatient settings), some outcome categories had to be removed since no concrete results were present in the full texts.

### 3.3 Systematic classification of the likelihood of conclusive evidence (research question 2)

#### 3.3.1 Criteria for the likelihood of conclusive evidence

To address research question 2, we defined an abstract-based scheme that classifies reviews as high, medium, low, or inconclusive with respect to the likelihood that the full text contains conclusive evidence. While evidence conclusiveness indicates the degree to which evidence can support a specific conclusion or outcome (285), it must be distinguished from evidence quality, which refers to the methodological rigor and reliability of the data (286), and evidence strength, which pertains to the magnitude and consistency of the observed effects (287). In line with Babić et al. (2022) (2), we found no standardized definition of evidence conclusiveness. We therefore relied on transparent, reproducible signals that are routinely reported in titles and abstracts and prioritize designs at the top of evidence hierarchies (288, 289), which increase power and precision (290).

The following signals were identified:

- Meta-analysis: explicit mention of a meta-analysis in title or abstract.
- Significant results: explicit linkage of the term “significant” to outcomes, including significant positive as well as no significant differences or benefits.
- Quantified results: presence of quantitative information in the abstract, from basic direction of effect counts or percentages to aggregated results of a meta-analysis.
- Inconclusiveness: explicit absence of evidence, missing overall direction, or substantial conflicting or contradictive effects.

Meta-analysis was prioritized because it was consistently reported across abstracts and present in 30.4% (76/250) of studies. Focus on randomized controlled trials was not used as a criterion because abstracts mentioned included study designs in only 68.0% (170/250).

Following Babić et al. (2), we conducted a thematic analysis of abstracts capturing quantitative and qualitative signals of effect direction, statistical significance, and conclusions. Details are provided in Supplementary Material 3.

Across abstracts, 32.8% (82/250) explicitly referenced significance, typically without distinguishing statistical from clinical significance. Quantitative reporting appeared in 46.0% (115/250), including 37 abstracts with details on significant effects. In 31 abstracts, quantification was limited to numbers or percentages of studies or outcomes by effect direction.

The qualitative assessment yielded the following themes: a) explicitly significant positive effects (66/250, 26.4%), b) clearly positive (140/250, 56.0%), c) vaguely positive (146/250, 58.4%), d) neutral with comparable effects or no differences (36/250, 14.4%), e) explicitly no significant differences or benefits (34/250, 13.6%), f) negative effects (13/250, 5.2%), and g) insufficient, inconclusive, or conflicting evidence (57/250, 22.8%). Multiple allocations occurred in 61.2% (153/250), mostly within the same overall direction (104/250, 41.6%) and or across different interventions or outcomes (71/250, 28.4%).

Table 7 provides an overview of the final classification rules and associated signals.

**Table 7:** Systematic classification of the likelihood of conclusive evidence based on data extracted from titles and abstracts.

Likelihood of Conclusive Evidence	Classification rules	Examples
High	Meta-Analysis AND multiple (>1) “significant” quantified results reported	A Meta-Analysis of Randomized Controlled Trials AND significantly improved wound healing rate (RR = 1.44, 95% CI = 1.16-1.80, *p* = 0.001) and reduced adverse events (RR = 0.52, 95% CI = 0.34–0.80, *p* = 0.003) (124)
Medium	Meta-Analysis with at least comparable results to usual care OR No meta-analysis, but at least one “significant” effect reported in >25% of relevant studies	Meta-analyses demonstrating long-term ulcer healing and mortality were not significantly different between telehealth and standard care groups (146); A narrative approach was used to synthesize the data due to the heterogeneous nature of the data. … The psychological status and clinical outcome measures were all significantly improved (191)
Low	No meta-analysis and no “significant” results. Evidence is mainly or only reported qualitatively. If quantitative results are reported, none is described as “significant”	Findings demonstrated limited evidence supporting the effectiveness of exercise delivered via telerehabilitation (26); The use of artificial intelligence for reviewing second-generation colon capsule endoscopy images is promising (157)
Inconclusive	No evidence at all OR insufficient for directional conclusion OR conflicting or contradictive effects despite positive signals elsewhere	Of 20 articles retained for full-text analysis, none reported any health economic evidence. (35); There is insufficient evidence in the existing literature concerning the real impact of AI or DSS … (160); Authors’ conclusions: There is no clear good quality evidence for or against using virtual reality … (85); Four of the studies indicated that electronic prescribing significantly increases initial medication adherence, while four of the studies suggested the opposite. The remaining two studies found no significant difference … (144)

#### 3.3.2 Validation of the accuracy of the classification of conclusive evidence

We conducted a rapid full-text review of 138 open-access articles to test the accuracy of the abstract-based classification. The sub-sample comprised 16 of 22 studies predicted as high (72.7%), 46 of 74 as medium (62.2%), 63 of 133 as low (47.4%), and 13 of 21 as inconclusive (61.9%).

Table 8 reports confirmation rates by class as well as re-classifications. Details are available in Supplementary Material 3.

**Table 8:** Accuracy of the classification of the likelihood of conclusive evidence, number of full texts (*N* = 138).

Likelihood of Conclusive Evidence	High	Medium	Low	Inconclusive
Predicted (*n*)	16	46	63	13
Confirmed % (*n*)	100.0% (16)	76.1% (35)	96.8% (61)	61.5% (8)
Not confirmed % (*n*)	0.0% (0)	23.9% (11)	3.2% (2)	38.5% (5)
Re-classification	n/a	6 → High 5 → Low	1 → Medium 1 → Inconclusive	5 → Low

n/a, not applicable.

High and low likelihood of conclusive evidence were predicted very well (100.0% and 96.8%) from abstracts, whereas inconclusiveness was least accurately predicted (61.5%).

All 16 studies predicted as *high* reported quantitative aggregated meta-analytic results with multiple significant effects.

Among 46 predicted as *medium*, 31 included meta-analyses, of which six showed more than one significant effect and were re-classified as high. Ten of the remaining 15 full texts quantified at least one significant effect in over 25% of relevant studies, while five were re-classified as low because they lacked quantitative substantiation.

Of 63 predicted as *low*, none reported meta-analyses or aggregated significant quantitative effects; eight noted at least one significant effect in more than 25% of relevant studies, yet only one met the quantitative substantiation required for re-classification to medium. While four full texts stated limited evidence, three concluded with a positive direction, and only one had to be re-classified as inconclusive.

Eight of 13 predicted as *inconclusive* met at least one criterion, and five were re-classified to low because the full texts provided a directional conclusion that was absent from the abstracts.

The practical implication is limited attrition of potentially relevant studies. The re-classification from medium to low represents about eleven percent (5/46) of discarded full-text reviews, while the shift from low to medium implies fewer than two percent (1/63) missed opportunities for conclusive evidence. Medium classifications would in practice still enter full-text screening if there were insufficient high-likelihood studies, and low or inconclusive classifications would typically be excluded.

### 3.4 The PACE4DHI framework

**Figure 2:**
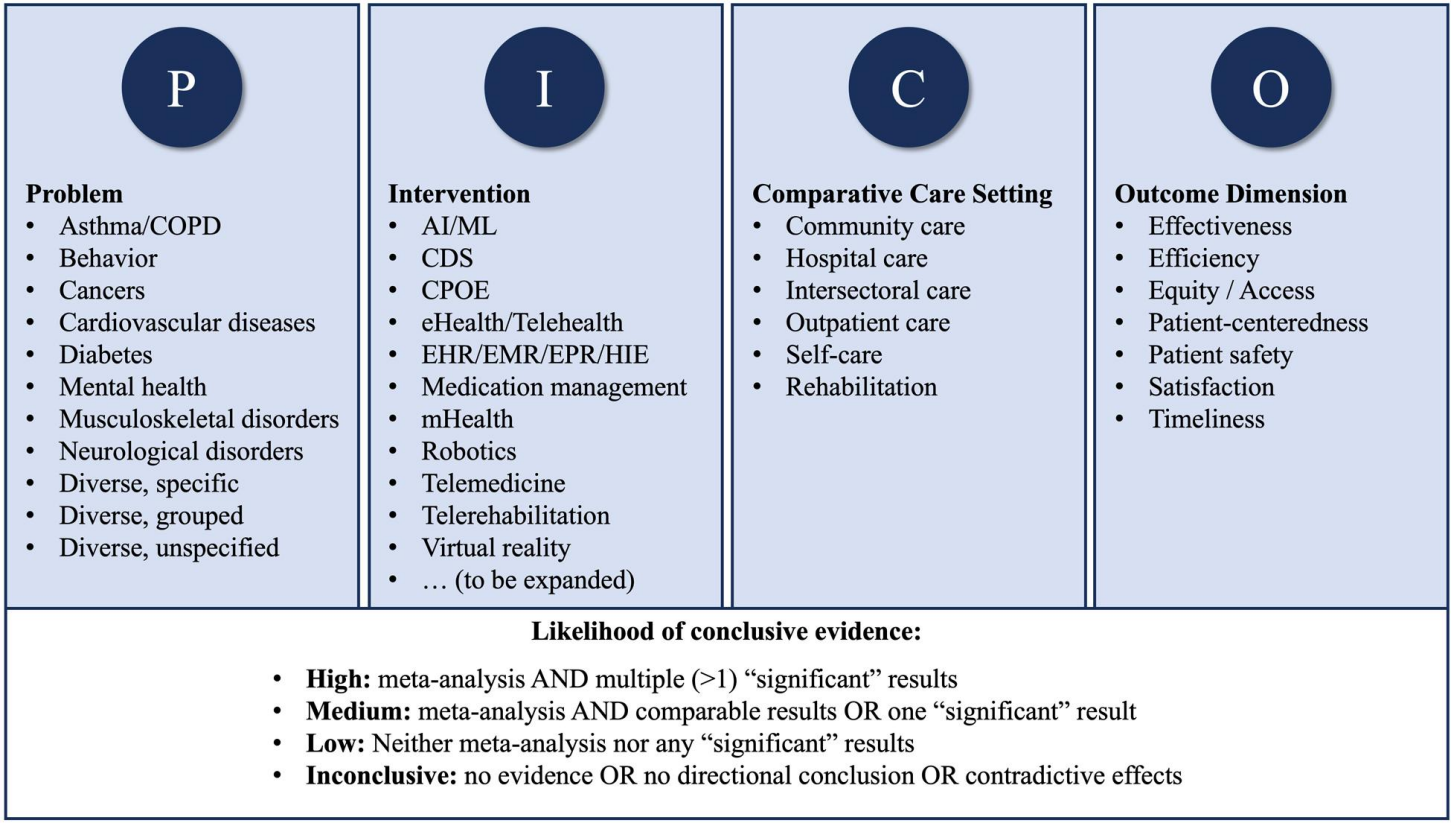
Overview of the PACE4DHI Framework.

Figure 2 provides an overview of the PACE4DHI framework resulting from the systematic categorization of PICO-Elements (Research Question 1) and the development and validation of criteria for the classification of the likelihood of conclusive evidence (Research Question 2).

## 4 Discussion

Despite the proliferation of digital health interventions (DHIs), it remains unclear which of these interventions have robust supporting evidence for which effects in different circumstances. This study seeks to bridge this gap by providing a framework to identify conclusive evidence on the outcomes and impacts of DHIs across diverse populations/problems, and care settings from titles and abstracts of systematic reviews. We achieved this by utilizing a structured categorization method that aligns with current research and established definitions. Our thematic analysis of titles and abstracts of systematic reviews and meta-analyses demonstrated that it is possible to define suitable categories at an effective level of abstraction, facilitating a clearer understanding of DHI impacts.

### 4.1 Principal results

We identified between 6 and 13 categories per PICO element through secondary research and thematic analysis to systematically structure current research evidence on DHIs during title and abstract screening (RQ 1).

We delineated 11 categories for the problem (P) category, which includes the six most prevalent chronic and noncommunicable diseases (NCDs), as well as behavioral risk factors and neurological disorders. Additionally, three categories cover diverse themes: highly specific conditions, undifferentiated disease groups (e.g., chronic diseases), or no specified condition at all. Further categories such as aged care and pediatric care may be considered but are likely to overlap with major identified themes.

For interventions (I), inductive thematic analysis yielded 11 primary categories and two additional technologies that were abstracted to potential categories. More categories may be added in the future, as research on additional interventions or technologies such as blockchain emerges.

The comparison (C) element is categorized by six key healthcare settings prioritized by policymakers.

For outcomes (O), we recommend utilizing the Six Domains of Healthcare Quality, complemented by satisfaction as an emerging concept.

In the validation, the PICO categories proposed in the framework and derived from the abstracts corresponded to the categories found in the full texts. However, there was a higher degree of accuracy for P and I than for C and O of the PICO scheme due to differences in the clarity and comprehensiveness of their description in the abstracts.

We identified and validated “meta-analysis”, “significance”, “quantification”, and “inconclusiveness” (RQ 2) as criteria to classify the likelihood of conclusive evidence for DHIs available in the full text during title and abstract screening. High and low likelihood of conclusive evidence were predicted very well (100.0% and 96.8%) from abstracts, whereas inconclusiveness was least accurately predicted (61.5%).

### 4.2 Limitations

#### 4.2.1 Sample size

A limitation of this study is the sample size of 250 abstracts, which represents about ten percent of eligible studies and provides a 95% confidence level that the actual value is within ±6% of the measured/surveyed value. This is considered sufficient, since our analysis aims for thematic saturation rather than statistical representativeness. The validation sample was limited to 138 open-access, full-text articles. However, with an error margin of ±5.6% and a 95% confidence level, this sample adequately confirmed that the categories and criteria are suitable for avoiding false positive or negative categorization and classification.

#### 4.2.2 Search period

The search period was confined to publications from 2011 to October 2023. An updated search strategy indicated a significant increase in publications, showing a 60.6% rise (from 12,608 to 20,246) when comparing search results from October 2023 to October 2025. Although excluding the most recent publications may alter the relative distribution of studies across existing categories (e.g., increasing focus on AI), it is not anticipated to result in the omission of entirely new emerging categories. However, the observed surge in the number of publications underscores the necessity for more efficient methods of evidence screening and synthesis.

#### 4.2.3 Focus on systematic reviews and meta-analyses

The framework was derived from an analysis of these types of review, while other common types, such as scoping reviews, were excluded. However, the framework's application is not limited to systematic reviews, and it can be used for any form of evidence synthesis or thematic evidence collection. During development, the framework's suitability for EGM was tested by manually creating a visual overview representing the intervention and outcome categories combined with the classification of evidence conclusiveness.

#### 4.2.4 Definition of comparison (C) as healthcare setting

Our definition of the comparator, focusing on the healthcare settings where DHIs are applied, diverges from the original PICO concept, which typically compares DHIs to alternative care practices or interventions. Our approach addresses limitations of the original concept within our framework's objectives and applicability to abstracts. Many systematic reviews lack a clear comparator and abstracts often do not specify one. By considering the healthcare settings where DHIs are used, we offer a broader “comparison” that helps decision-makers by identifying where DHIs are most beneficial.

#### 4.2.5 Risk of incomplete categorization of PICO elements

While the proposed PICO categories effectively provided a framework for structuring problems (P), comparative settings (C), and outcomes (O) across abstracts from systematic reviews and meta-analyses on DHI impacts, the interventions (I) may require further expansion to accommodate additional digital health interventions as they emerge.

#### 4.2.6 Risk of bias due to lack of consideration of evidence quality or strength in evidence classification

Our methodology emphasizes systematic reviews and meta-analyses to mitigate bias associated with varying evidence levels. The disparate ranking, grading, and rating systems currently in use complicate the consistent assessment of evidence quality and strength and are not suited for efficient screening, since they are inconsistently reported in the abstracts.

#### 4.2.7 Internal validation

Our validation analysis represents an internal validation based on an availability-based subsample of 138 open-access full texts. We acknowledge the lack of an independent validation sample as a potential source of selection bias. However, this risk appears neglectable, with the validation sample representing 55.2% of the development sample. While the internal validation confirmed suitability and informed immediate application, we encourage external validation on an independent sample by peer researchers wishing to implement the PACE4DHI framework. *Risk of future manipulation in abstracts:* There is a potential risk for bias if authors deliberately craft abstracts to suggest a higher likelihood of conclusive evidence, should the framework become widely adopted. Despite this, we consider the risk manageable, as the use of the framework can simultaneously enhance research question formulation and the articulation of findings, thereby potentially improving the overall quality of abstracts and research.

### 4.3 Conclusions

#### 4.3.1 Overview

The study successfully developed and validated the PACE4DHI framework to systematically categorize and classify existing evidence for digital health interventions (DHIs) using the PICO approach. It offers an efficient structured means for abstract screening and is intended to facilitate continuous evidence and gap mapping, providing a practical foundation for identifying conclusive evidence of achievable impacts and research gaps in digital health.

#### 4.3.2 PICO-based categorization

The research successfully identified categories aligned with current research as well as established schemes and definitions and demonstrated that the framework could effectively organize research evidence from systematic reviews and meta-analyses along PICO. This organization aids in elucidating the diverse impacts of DHIs, given the wide range of populations, interventions, comparisons, and outcomes.

#### 4.3.3 Criteria for assessing evidence

The study identified and validated four key criteria for determining the likelihood of conclusive evidence being present in the full texts based on information that can be retrieved from abstracts: presence of a meta-analysis, the mention of significance related to results, result quantification, and indication of inconclusiveness. This approach enhances the probability to identify and retrieve all relevant evidence and can thus improve the evaluation of achievable DHIs' impacts.

#### 4.3.4 Practical applications

The framework is intended to be used by researchers in a) formulating research questions aligned with current research and established definitions, b) informing comprehensive search strategies by providing associated search terms, c) driving efficiency in abstract screening of an ever-increasing body of studies, d) training artificial intelligence tools that support screening, extraction and synthesis from systematic reviews and meta-analyses, e) enabling subsequent continuous EGM guiding future research priorities and aiding decision-makers in evaluating the potential benefits of DHIs.

#### 4.3.5 Implications

The PACE4DHI framework strengthens the strategic approach to building a comprehensive evidence base in the digital health sector. The application of the framework can help decision-makers at different levels (organization, politics, research) a) to inform digitization strategies more easily, b) to design implementation plans in an evidence-based manner, c) to identify research gaps more quickly and systematically. Further and ongoing refinement of terms associated with the categories and criteria is warranted to adapt the framework for use with advanced artificial intelligence tools, which can automate and enhance the research process in this rapidly evolving field.

## Data Availability

The original contributions presented in the study are included in the article/Supplementary Material, further inquiries can be directed to the corresponding author.
