# Supplementary material for: PICO-based assessment and categorization of evidence for digital health interventions: an inductive framework development
Source: Front Digit Health. 2026 Feb 18;8:1755598. doi: 10.3389/fdgth.2026.1755598 (PMC12957232; doi:10.3389/fdgth.2026.1755598)
Supplement: Supplementary file 1 [file Supplementaryfile1.docx]

Supplementary Material 1 - Search terms

# Interventions

1. MeSH terms: decision support systems, clinical ; medical records systems, computerized ; reminder systems ; health records, personal ; telemedicine ; hospital information systems ; nursing informatics ; health information interoperability ; consumer health informatics ; Artificial Intelligence ; Big Data ; Radiology Information Systems
2. Search terms in titles and abstracts: "health information technolog*" ; "clinical information system*" ; "clinical decision support" ; "electronic health record*" ; "electronic medical record*" ; "health smart card*" ; "health information exchange" ; "reminder system*" ; "personal health record*" ; "patient portal*" ; "telemedicine" ; "mhealth" ; "ehealth" ; "telehealth" ; "mobile health" ; "hospital information system*" ; "computerized provider order entry" ; "closed loop medic*" ; "nursing informatic*" ; "nursing information system*" ; "interoperab*" ; "consumer health informatic*" ; "CDSS" ; "CPOE" ; "EHR" ; "EMR" ; "artificial intelligence" ; "blockchain*" ; "distributed ledger*" ; "e-health" ; "Radiology Information System*" ; "Picture Archiving and communication system*" ; "PACS"

# Outcomes

1. MeSH terms: quality improvement ; meaningful use ; quality of health care ; outcome and process assessment, health care ; efficiency ; costs and cost analysis ; patient safety ; medical errors ; medication errors ; empowerment ; patient acceptance of health care ; digital divide ; learning health system ; Health Services Accessibility ; Health Equity
2. Search terms in titles and abstracts: "quality improvement*" ; "meaningful use" ; "quality of health care" ; "quality of healthcare" ; "health care quality" ; "healthcare quality" ; "quality of care" ; "care quality" ; "patient outcome*" ; "treatment outcome*" ; "patient relevant outcome*" ; "effectiveness" ; "efficiency" ; "productivity" ; "cost-effectiveness" ; "cost effectiveness" ; "cost benefit*" ; "cost-benefit*" ; "cost saving*" ; "patient safety" ; "medical error*" ; "medication error*" ; "prescription error*" ; "patient acceptance of health care" ; "health care utilization" ; "healthcare utilization" ; "patient compliance" ; "medication adherence" ; "drug adherence" ; "medication compliance" ; "patient participation" ; "patient engagement" ; "digital divide" ; "health equity" ; "learning health system*" ; "access to health" ; "accessibility" ; "prescribing error*" ; "unintended consequence*" ; "adverse effect*" ; "outcome*"
