# Supplementary material for: PICO-based assessment and categorization of evidence for digital health interventions: an inductive framework development
Source: Front Digit Health. 2026 Feb 18;8:1755598. doi: 10.3389/fdgth.2026.1755598 (PMC12957232; doi:10.3389/fdgth.2026.1755598)
Supplement: Supplementary file 4 [file Supplementaryfile4.docx]

Supplementary Material 4 - Thematic categories of PICO-elements including references

Table 2. Thematic categories of PICO-element P - Problem, number (n) and percentage (%) of N = 250 abstracts, multiple allocations possible.

| **#** | **Category** | **Description and examples** | **n (%)** |
| --- | --- | --- | --- |
| 1. | Cardiovascular diseases (CVDs) | Includes heart failure (26–30), high blood pressure / hypertension (29,31–33), stroke (34,35), ischemic heart disease (29), coronary artery disease (36), cardiac and vascular surgery (37,38), cardiac rehabilitation (39). | 41 (16.4) |
| 2. | Diabetes | Any studies related to diabetes in general (40,41), specific types (mellitus, type 2) (42–45), diabetes-related conditions (46–51), and diabetes as a risk factor (40,41) | 30 (12.0) |
| 3. | Behavior | Concerned with changing lifestyle behaviors (52) including physical activity (53–58), diet and weight (53,57,59,60), smoking cessation (53,61,62), contraception (63), intimate partner violence (64), etc. | 28 (11.2) |
| 4. | Cancers | Encompasses various forms of cancers (65–69), cancer screening and detection (70,71), cancer-related conditions (e.g., polyps (72), hematological conditions (73)), cancer treatment (74–76), burden (77–79), and aftercare (80,81). | 26 (10.4) |
| 5. | Mental health | Encompasses various mental health issues (82–84) and mental illnesses (85), including depression (48,86–90), suicidal ideation (91,92), insomnia (93,94), caregiver stress (95), etc. | 25 (10.0) |
| 6. | Neurological disorders | Any studies on disorders that affect the brain, spinal cord or nerves, and related interventions, including dementia (96–98), stroke (34,35,99), Alzheimer's disease (97,100), autism spectrum disorders (101,102), spinal cord injury (103,104), neurosurgery (105), neurorehabilitation (106). | 25 (10.0) |
| 7. | Musculoskeletal disorders | Any studies on disorders that affect muscles, tendons, joints and cartilage, including rheumatic diseases (107–109) such as osteoarthritis (110,111), related pain (112–114), and rehabilitation following arthroplasty (107–109). | 10 (4.0) |
| 8. | Chronic pulmonary diseases | Particular focus on asthma and COPD (115–118) or among the identified conditions targeted by the DHI (26,30,119). | 8 (3.2) |
| 9. | Diverse groups of conditions | Encompasses unspecified groups of conditions such as chronic diseases (120–122), noncommunicable diseases (123), chronic wounds (124), digestive diseases (125), sexually transmitted infections (126,127), skin neglected tropical diseases (128), and medical specialties, e.g., pregnancy and obstetrics (129–131), pediatrics (132–135), palliative care (132,136), aged care (137), anesthesia (138), dermatology (128,139), intensive care (140), urology (141), etc. | 40 (16.0) |
| 10. | Diverse, unspecified | Studies with no specific focus on any medical condition or applicable across all health conditions (142); often related to medication (143–149) | 27 (10.8) |
| 11. | Diverse specific conditions | Includes specific diseases (e.g., HIV (150,151), COVID-19 (152,153), Chronic Kidney Disease (154), Pulmonary Tuberculosis (155), Sickle Cell Disease (156)), diagnostic, therapeutic or surgical interventions (e.g., Colon Capsule Endoscopy (157), Anticoagulation Therapy (158), renal transplant (159)) and clinical objectives (e.g., prediction of hospital-acquired pressure injuries (160)) | 17 (6.8) |
|  | Multiple Category Allocation | Studies may be allocated to more than one category for stroke as CVD and neurological disorder (34,35,161–164), for comorbidities such as mental health issues associated with cancer or diabetes or neurological disorders (48,77,78,165) or hypertension in pregnancy (33), and for unspecified or wider research scope (e.g., chronic diseases, NCDs) but focused findings (e.g., diabetes, CVDs) (26,30,123,166) | 23 (9.2) |

Table 3. Thematic categories of PICO-element I - Intervention, number (n) and percentage (%) of N = 250 abstracts, multiple allocations possible.

| **#** | **Category** | **Description and examples** | **n and %** |
| --- | --- | --- | --- |
| 1. | Telemedicine | Refers to remote patient interaction with clinical providers for assessment (180), diagnosis (181), consultation (182,183), therapy (34,156,184) and management, including telemonitoring (27–30,33,46,180,182,184), and specialty forms such as telepsychiatry (82,185), teledermatology (128), telerheumatology (181), telenursing (30), telestroke (161), teleophthalmology (186), and virtual urology clinics (141). | 66 (26.4) |
| 2. | eHealth/Telehealth | Includes remote and online web- or internet-based interventions via home computers or mobile devices without or with interaction (i.e., telehealth (35,57,84,115,132,136,150)), and with a focus on behavior change (52,53,156,180), incl. CBT (48,93,165), health education (64,98,130,187), counseling (31,63,184,188), psychological support (92,98,189,190), and support for self-management (48,63,154,177). | 64 (25.6) |
| 3. | mHealth | Focuses on self-care and self-management without health provider interaction, involving smartphone applications (37,56,58,80,97,122,123,156,184,191,192), wearables (41,54–56,192–194) and sensors (55,195). | 60 (24.0) |
| 4. | Artificial Intelligence | Encompasses studies on AI techniques, including deep learning and machine learning (67,91,196,197), used in diagnostics (72,113,198), especially imaging and radiomics (67,155,157,199–201), detection (71,202), prognosis and prediction (65,91,160,203–205), and treatment delivery, e.g., by AI-powered chatbots (90,206). | 30 (12.0) |
| 5. | Telerehabilitation | Concerns the delivery of rehabilitative programs and exercise interventions via videoconferencing (26,120,207,208), apps (106,107,112,209), and virtual reality rehabilitation (99,109,210). Includes cardiac rehabilitation (29,36,39,209) and neurorehabilitation (106). | 29 (11.6) |
| 6. | Medication Management | Encompasses a variety of technologies and processes, from medication reconciliation (143,145), through electronic prescribing (75,144,147,211) and medication administration (149), to supporting adherence (212–214). | 14 (5.6) |
| 7. | EHR/EMR | Covers Electronic Health Records (EHRs) (60,83,215–217), Electronic Medical Records (EMRs) (218,219), Electronic Patient Records (EPRs) (220), and Health Information Exchange (HIE) between them (215,221). | 13 (5.2) |
| 8. | Clinical Decision Support | Relates to systems aiding clinical decision-making, including prediction (137,160) and evidence-based treatment (89,222), also using AI and ML (160,163,196,205). | 13 (5.2) |
| 9. | Robotics | Encompasses any use of robotic technologies in healthcare, including surgeries, treatment, and logistics, e.g., robot-assisted surgery (76,134), robot-assisted gait training (103,104), social or companion robots (223). | 6 (2.4) |
| 10. | Virtual Reality | Encompasses a wide range of applications such as simulation, visualization, training, teaching, learning, etc., e.g., utilized for treatment compliance (85) and for rehabilitation training (99,109,210). | 4 (1.6) |
| 11. | CPOE | Computerized Physician Order Entry systems (224–226). | 3 (1.2) |
| 12. | Imaging | The examples pertain to Picture Archiving and Communication Systems (140) and to wound assessment imaging and monitoring (50). | 2 (0.8) |
| 13. | Patient Portal | Online applications that provide patients access to information and allow them to interact with their healthcare providers, for example via web-based medical appointment systems (227). | 1 (0.4) |
|  | Multiple Category Allocations | Most often pertaining to intersections between mHealth, eHealth/telehealth, telemedicine, and telerehabilitation (29,62,63,80,115,150,156,184,228–231), along with combinations relating to CDS and AI (160,163,196,205) or CPOE (224). | 44 (17.6) |

Table 4. Thematic categories of PICO-element C - Comparison, number (n) and percentage (%) of N = 250 abstracts, multiple allocations possible.

| **#** | **Category** | **Description and examples** | **n and %** |
| --- | --- | --- | --- |
| 1. | Outpatient Care | Explicitly encompasses primary care (83,166,198,237), ambulatory care (211,218), outpatient setting (144,238), non-urgent health care services / practices (227), and a variety of typical outpatient specialist care and mental health services (33,63,72,73,82,122,129,139,141,165,180–182,186,239,240). Implicitly referred to by the primary management of many chronic conditions, e.g., diabetes (42,43,46,47,230,241,242), blood pressure (31,32,231,243) and cardiovascular diseases (27–29,244,245), chronic pulmonary disease (115,116,148), chronic wounds (124). | 83 (33.2) |
| 2. | Self-care and Self-management | Focuses on interventions to improve self-care and self-management skills in various conditions (41,92,114,117,154,191,192,246–250), self-support for lifestyle behavior changes (55,59,176,206,251) and for informal caregivers (77,96,98,100), typically without provider involvement (40,52,53,55,56,61,62,80,252), and often linked to mHealth (97,123,213). | 66 (26.4) |
| 3. | Community-based Care | Includes behavioral and psychosocial specialists (48,53,57,59,95,98,101,189,253,254), physical therapy (194,208), home-based, community and residential care settings (30,54,137), long-term care (255), palliative care (132,136), and preventive health care (204,245). | 35 (14.0) |
| 4. | Intersectoral | Reflects interventions that transcend care settings, such as Health Information Exchange (215,221), medical data processing in EHRs (e.g., for screening) (216,217), medical interpretation (256), a multidisciplinary care approach (113,257,258). Implicitly pertains to cancer diagnostics and treatment (65,68,69,75,199,200,259). | 32 (12.8) |
| 5. | Hospitals | Refers to hospital inpatient care (34,143,147,160,203), acute care (161,196,260), surgery-related interventions (38,76,105,134,135,138,145,205,261,262), peri- and neonatal care (131,226,263), intensive care (140), emergency department visits (237,264); Implicitly pertains to CPOE or CDSS (138,222,226,265). | 31 (12.4) |
| 6. | Rehabilitation | Covers explicit comparisons with conventional rehabilitation (107–112,142,266,267), in various rehabilitation settings, specifically cardiac rehabilitation (36,39,209,258,268), neurorehabilitation (99,103,106,162,164,180), and cancer aftercare (66,81). | 28 (11.2) |
|  | Multiple Category Allocation | Studies applying to two or more settings, most often self-care and community care (48,53,54,58,59,98,269,270), inpatient hospital and outpatient care (131,146,222,237,263,265) or outpatient care with any other settings (58,87,99,180,184,231,245). | 23 (9.2%) |

Table 5. Thematic categories of PICO-element O - Outcome, number (n) and percentage (%) of N = 250 abstracts, multiple allocations possible.

| **#** | **Category** | **Description and examples** | **n and %** |
| --- | --- | --- | --- |
| 1. | Effectiveness | Encompasses a wide range of measurable clinical outcomes including symptoms (e.g. pain, depression) (36,48,81,84,95,98,110–112,125,132,222), disease control indicators (e.g. blood pressure) (29,31,32,36,42,138,154,218), mortality (27,28,34,46,124,182), biochemical indicators (e.g. glucose, HbA1c) (42–44,47,123,246), anthropometric indicators (e.g. BMI, weight) (29,36,42,47), physical function / exercise capacity (e.g., speed, balance, etc.) (103,106,109,111), cognitive function (e.g. memory) (81,85,99), pulmonary function (115,118,152), healing rate (49,124,183), obstetric outcomes (129,131,263), objective sleep quality (93,94), and process of care measures (e.g., risk screening rates) (70,89,121,148,154,176,217,218,255,274). | 151 (60.4) |
| 2. | Patient-centeredness | Any outcomes related to an interventions responsiveness to individual patient preferences, needs, and values (275), with a focus on psychosocial and patient-centered implementation outcomes (44), including health‐related quality of life (HRQoL) and well-being (26,30,36,37,42,48,80,81,97,105,110,112,115,120,125,132,154,192,276), lifestyle changes (e.g. diet, physical activity, abstinence, contraception) (52–55,58,61–63,80,123,246), activities of daily living (99,210,277), emotional / social skills and needs (81,85,101,217), patient engagement (37,189,220,270,278) and treatment adherence / compliance (63,85,115,125,144,150,154), education (187,263,279), self-management / -efficacy (156,228,247,280,281), informal caregiver support (77,96,98), family functioning (133), care coordination (83), avoiding travel time and costs (38,51,249), etc.. | 119 (47.6) |
| 3. | Efficiency | Covers cost-effectiveness (42,46,51,57,82,86,139,184,185,240,253), costs and cost-savings (44,49,108,135,141,142,185,208,282), healthcare resource utilization (30,37,138,179,192,217,225,263,283), e.g., emergency department visits, test consumption, hospitalizations, length of stay, and readmissions (27,28,33,115,138,217,228,260,264), efficiency of work practices, productivity and time savings (42,75,140,180,200,215), patient attendance and no-show rates (176,227,284). | 63 (25.2) |
| 4 | Patient Safety | Focuses on avoiding harm, including improved diagnostic decision making and accuracy in diagnoses (59,68,69,72,113,155,179,198,199,201,218), improved medication safety (37,75,143,145–147,149,179,255), prophylaxis, prediction and prevention of adverse events (36,124,138,160,178,211), complications (74,141,197,204,205,218), clinical deterioration and exacerbation (118,203,237), adverse pregnancy outcomes (33,45,131), procedural errors (196), infections (153,261), pressure injuries (160), thromboembolic events (158), hypoglycemia (47), falls (100,137), suicide (91), etc., and provider adherence to guidelines and protocols (48,75,121,225). | 62 (24.8) |
| 5. | Satisfaction | Comprises user satisfaction among patients (37,80,132,141,154,156) and healthcare providers (75,126,256), related to satisfaction with the treatment (66,85,253) and acceptability/acceptance (75,85,88,132,154,156,243) of the intervention. | 37 (14.8) |
| 6. | Timeliness | Relates to decreased waiting time (227,253), onset-to-door (OTD) duration (161), turnaround times (225), earlier diagnosis (198), timely intervention (37), time to clinical action (140); also immediate access to special services such as medical interpretation (256). | 8 (3.2) |
| 7. | Equitable Access to Care | Involves improved access to health care providers including specialist care (83,128,182), services and resources (217,283), but no study in the sample specifically reported on equitable access. | 6 (2.4) |
|  | Multiple Category Allocation | Multiple outcomes are commonly reported on two or more (up to five) outcome dimensions. | 131 (52.4) |
